# Do you look for information about dementia? Knowledge of cognitive impairment in older people among their relatives

**DOI:** 10.1590/1980-57642021dn15-020013

**Published:** 2021

**Authors:** Mariel Carolina Montiel-Aponte, Paulo Henrique Ferreira Bertolucci

**Affiliations:** 1 Postgraduate Program in Neurology and Neurosciences, Escola Paulista de Medicina, Universidade Federal de São Paulo - São Paulo, SP, Brazil.; 2 Escola Paulista de Medicina, Universidade Federal de São Paulo - São Paulo, SP, Brazil.; 3 Behavioral Neurology Outpatient Clinic, Hospital São Paulo - São Paulo, SP, Brazil.

**Keywords:** dementia, cognitive impairment, caregivers, relatives, conhecimento, comprometimento cognitivo, cuidadores, familiares

## Abstract

Relatives and caregivers receive little information and have poor knowledge about cognitive impairment and dementia.

**Objective:**

This study aimed to identify beliefs about cognitive impairment and aging among people who are in contact with older people with and without cognitive impairment, hypothesizing that the fact of being a close relative influences or modifies these beliefs.

**Methods:**

Seventy-eight participants were classified into two groups; group 1: relatives of patients with cognitive impairment or dementia from a behavioral neurology outpatient clinic (n_1_=48); and group 2: relatives of patients without objective cognitive impairment from different services of a geriatric outpatient clinic (n_2_=30). All subjects were asked to answer a questionnaire containing single choice and true/false questions about causes and risk factors for dementia.

**Results:**

Participants were mainly females and first-degree relatives. No statistical differences were observed for age, schooling, or follow-up time between groups. Participants recognized Alzheimer’s disease as the main cause of memory loss in older adults (group 1=34 *vs.* group 2=15); when asking about sources of information about cognitive impairment, the three more common answers were *doctors and health professionals*, *Internet*, and *journals/books*. Group 1 got higher scores on questions about causes and risk factors for dementia, but no statistical differences were found.

**Conclusions:**

Dementia literacy is low even among the people in contact with this syndrome; caring for someone with dementia changes the concepts about memory and aging but only in a small proportion. Educational strategies to deal with misinformation can help to control risk factors and reduce the incidence of dementia.

## RESUMO.

Parentes e cuidadores de idosos recebem pouca informação e têm pouco conhecimento sobre demência.

**Objetivo:**

O presente estudo teve como objetivo identificar crenças sobre déficit cognitivo e envelhecimento nas pessoas que estão em contato com idosos com e sem comprometimento cognitivo, estimando que ser familiar próximo influencia ou modifica essas crenças.

**Métodos:**

Setenta e oito (78) participantes foram classificados em dois grupos. Grupo 1: parentes de idosos com comprometimento cognitivo ou demência em acompanhamento em um ambulatório de neurologia do comportamento (n_1_=48); grupo 2: parentes de idosos sem déficit cognitivo objetivo em acompanhamento em diferentes consultas dos ambulatórios de geriatria (n_2_=30). Os participantes responderam a um questionário com perguntas de escolha simples e verdadeiro/falso sobre causas e fatores de risco para demência.

**Resultados:**

A maioria dos participantes foi do sexo feminino, principalmente filhos e cônjuges. Não houve diferenças significativas para idade, escolaridade ou tempo de acompanhamento. Os participantes reconheceram a doença de Alzheimer como a causa principal de déficits de memória em idosos (grupo 1=34 *versus* grupo 2=15). Quando questionadas as principais fontes para se informar sobre déficits cognitivos, as três respostas mais comuns foram *Doutores e profissionais de* saúde*, Internet* e *Revistas/Livros*. Nas questões sobre causas e fatores de risco para demência, o grupo 1 teve mais respostas corretas, porém sem diferenças estatísticas.

**Conclusões:**

O grau de conhecimento das pessoas que estão em contato com essa síndrome ainda é baixo. Cuidar de alguém com demência muda as crenças sobre memória e envelhecimento, mas apenas em uma pequena proporção. Estratégias educativas para lidar com a desinformação são necessárias para ajudar no controle de fatores de risco e reduzir a incidência da demência.

## INTRODUCTION

Life expectancy has increased significantly in the last decades according to data from the Global Health Observatory of the World Health Organization.^1^ In 2019,^1^ the average life expectancy at birth was 72.2 years for the global population; however, the average of healthy life years was just 63.3 years, indicating that these extra years come together with a higher prevalence of chronic diseases, with dementia being one of the ten causes of disability and dependency in older people worldwide.^2^ ^,^ ^3^ While a total of 35.6 million people were living with dementia in 2010, projections for 2030 show that this number could be around 82 million, reaching 152 million in 2050.^3^ This scenario indicates a serious public health problem and, considering the existing options of treatment, prevention is the main measure that could be taken to reduce its impacts.

Dementia causes disability and dependency and brings a significant burden for relatives and caregivers.^4^ ^,^ ^5^ ^,^ ^6^ Spouses, children, close friends, and others play a crucial role in dementia management, as they guarantee adherence to medical treatment and provide support and assistance for the patients in all their activities and needs to continue living with quality and dignity.^6^ Their contributions go beyond economic costs, as there is a complex interaction between people with dementia and caregivers involving social, emotional, and cognitive and physical health dimensions.^6^ ^,^ ^7^ ^,^ ^8^ ^,^ ^9^

Although most relatives and caregivers express their caring experience as something positive,^8^ many recognize that they receive little support and have poor knowledge about dementia, which makes it difficult to plan and understand the patient’s future needs during the course of the disease.^6^ ^,^ ^7^ Caregivers report lack of preparedness to provide care, and they tend to manifest stress, burden, mood disorders and feelings of lack of social networks for support at some point of the dementia journey.^9^ ^,^ ^10^ ^,^ ^11^

The number of caregivers and people with dementia receiving specific types of professional support is low, and informal caregivers express their need for additional professional support.^7^ ^,^ ^8^ ^,^ ^12^ However, this kind of assistance doesn’t seem to improve throughout the course of dementia.^7^ ^,^ ^8^ ^,^ ^12^ ^,^ ^13^

In Brazil, some studies have tried to identify knowledge and beliefs about dementia among relatives, caregivers, and the general population.^5^ ^,^ ^14^ A study performed in 2018^14^ adapted the Dementia Knowledge Assessment Tool-2 to Brazilian Portuguese and applied it to a sample of 30 caregivers of patients with dementia; the mean of correct answers was 15/21 (±2.5). Lower scores were related to higher Zaria Burden Interview and lower Functional Activities Questionnaire (FAQ-P) results. It reflects the importance of planning educational strategies, because misinformation is associated with burden and psychiatric symptoms in caregivers and relatives.^7^ ^,^ ^8^

Supporting informal caregivers is a key point to guaranteeing quality of life for people with dementia and protecting caregivers’ physical and mental health as well.^3^ ^,^ ^9^ ^,^ ^15^ ^,^ ^16^ Providing an effective support for caregivers is important for them to know the problems, needs, questions, perceptions, and experiences associated with dementia, always keeping in mind that these may change during the course of the disease.^1^ ^,^ ^3^ ^,^ ^6^ ^,^ ^7^

This study aimed to identify the beliefs about cognitive impairment and aging among relatives of older adults with and without cognitive impairment, as well as to determine which sources are important for them to get informed about cognitive impairment. Identifying these beliefs enables to planning and design of educational interventions to increase dementia literacy among the relatives of these patients and also to educate the general population.

## METHODS

The study was conducted between November 2019 and August 2020, at the Behavioral Neurology Outpatient Clinic and at the Cardiology, Musculoskeletal and Longevity Outpatient Clinics of the Geriatrics and Gerontology Discipline of São Paulo Hospital, Universidade Federal de São Paulo - Escola Paulista de Medicina (UNIFESP-EPM).

This study was approved by the Research and Ethics Committee of the UNIFESP-EPM (number: 3.736.135; CEP/UNIFESP No. 1221/2019).

This study was partially supported by the Coordenação de Aperfeiçoamento de Pessoal de Nível Superior - Brasil (CAPES) - Finance Code 001.

### Participants

Relatives and/or caregivers of patients being monitored at these outpatient clinics were invited to participate and were classified into two groups:

- Group 1: Relatives of patients with dementia or mild cognitive impairment from the Behavioral Neurology Outpatient Clinic (n_1_=48).
- Group 2: Relatives/caregivers of patients without objective cognitive impairment from the Cardiology, Musculoskeletal and Longevity Geriatrics Outpatient Clinic (n_2_=30).

For inclusion in group 1, the participants were the patient’s relatives, aged 18 years or older, and had attended at least two previous medical consultations, considering this period as the minimum time to receive clinical guidance about the diagnosis of the patient. The same inclusion criteria were applied for group 2, and the absence of objective cognitive impairment was verified consulting the patient’s medical records of cognitive assessment. All volunteers read and signed an informed consent form before the interview.

### Materials and procedures

Demographic data of the participants and patients were collected, and a structured questionnaire (Attachment 1) was then presented to each participant. The questionnaire had one single-choice question and five true/false questions about the causes of cognitive impairment and associated risk factors. Instructions were read and explained, and the participants filled out the questionnaires with minimal assistance, after ensuring they clearly understood what was requested.

### Statistical analysis

The collected data were descriptively analyzed. For categorical variables, the absolute and relative frequencies were presented as proportions. For numerical variables, the summary measures (mean and standard deviation [SD]) were presented. The confidence intervals for proportions were calculated with 95% confidence intervals (95%CI) to investigate the differences between the groups. The Z-test for proportions was used to verify differences between the groups in nominal variables expressed as proportions. The Student’s t-test for two independent samples was performed to identify differences in numerical variables between the groups.

## RESULTS

Seventy-eight relatives/caregivers were interviewed and classified into two groups, as described. They were mainly female (n_1_:N_1_=37; proportion=0.77 VS n_2_:N_2_=26; proportion=0.87), and almost all were first-degree relatives (i.e., child, spouse, or sibling) in both groups. In group 1, a total of 23 participants (proportion=0.48) described themselves as family caregivers, as they assisted the patients in many activities of daily living. In group 2, just 2 participants (proportion=0.06) were private caregivers, and they described themselves as non-relative person paid to visit the patients at home and help them in some activities such as housekeeping and for companionship as well. This result had statistical significance (p≤0.0001), showing that older adults with dementia need more assistance and care than older adults without cognitive impairment. Other demographic characteristics are shown in Table 1.

**Table 1.** Demographic characteristics of relatives/caregivers.

	Group 1 (N_1_=48)		Group 2 (N_2_=30)		Sig.*
Gender	n_1_:N_1_	proportion	n_2_:N_2_	proportion	
Female	37	0.77	26	0.87	0.27
Profession/occupation related to health care	8	0.17	3	0.1	0.39
Relationship					
Child	39	0.81	18	0.6	0.04**
Spouse	8	0.17	3	0.1	0.39
Sibling	0	0	2	0.07	0.06
Other	1	0.02	7	0.23	<0.01**
Caregivers	23	0.48	2	0.06	<0.001**
Have you met someone suffering from dementia? (Group 2 participants)	-	-	20	0.67	X
Optimism about the patient’s clinical condition/dementia	28	0.58	11	0.37	0.07
Belief in God	47	0.98	30	1	0.43

n_1:_N_1_: number of participants in group 1; n_2_:N_2_: number of participants in group 2; proportion: proportion in each group; *Z-test for proportion results; **significant results.

For group 1, the mean age was 52.31 (±13.14) years, and the mean number of years of schooling was 12.73 (±4.89); their relatives with cognitive impairment and dementia had a mean caring time of 4.54 (±4.34) years. For group 2, the mean age was 55.10 (±12.06) years old, and the mean of years of schooling was 13.07 (±4.40); their relatives had a mean caring time of 6.47 (±4.35) years (Table 2). No statistical differences were found when comparing the two groups.

**Table 2.** Summary measures: age, years of schooling, and follow-up time.

	Group 1 (N_1_=48)	Group 2 (N_2_=30)	Sig.*
	Mean (±SD)	Mean (±SD)	
Age	52.31 (±13.14)	55.10 (±12.06)	0.67
Years of schooling	12.73 (±4.89)	13.07 (± 4.40)	0.75
Follow-up time	4.54 (±4.34)	6.47 (±4.35)	0.11

SD: standard deviation; *Student’s t-test for two independent samples.

### Interest in understanding cognitive problems

We explored the differences between the groups regarding the self-initiative of the participants to be informed about dementia. The confidence intervals for proportions (n_1_:N_1_=31; proportion=0.65 [0.52‒0.78] *vs.* n_2_:N_2_=10; proportion=0.33 [0.24‒0.42]) did not show overlap, whereas the results of the Z-test for proportions showed statistical significance (p≤0.01). This might mean that the relatives of patients with cognitive impairment have more self-initiative to look for information about dementia (Table 3). Considering that being a caregiver might be a characteristic explaining why individuals in group 1 have more self-initiative to be informed about dementia, we subclassified group 1 into *1.1. Relatives and caregivers* (n=23) and *1.2. Non-caregiver relatives* (n=25), and investigated the possible differences using a Z-test for proportions (group 1.1: n_1.1_=11, proportion=0.47 *vs.* group 1.2: n_1.2_=20, proportion=0.8; p*=*0.02). Curiously, the results showed that the non-caregiver relatives looked for information more frequently.

**Table 3.** Questions about cognitive impairment and dementia: correct answer proportion.

Have you looked for information about dementia?							
Answers	Group 1 (N_1_=48)			Group 2 (N_2_=30)			Sig.*
	n_1_:N_1_	proportion	95%CI	n_2_:N_2_	proportion	95%CI	
Yes	31	0.65	[0.52-0.78]	10	0.33	[0.24-0.42]	<0.01**
Would you like to participate in an educational program about cognitive impairment and aging?							
Yes	43	0.89	[0.74-1.04]	26	0.87	[0.68-1.06]	0.79
Which of the following is the most common cause of memory loss in people over 65 years old?							
Normal aging	12	0.25	[0.13-0.37]	12	0.4	[0.32-0.68]	0.16
Arterial thickening	2	0.04	[-0.02-0.1]	3	0.1	[-0.01-0.21]	0.29
Alzheimer’s disease	34	0.71	[0.57-0.85]	15	0.5	[0.22-0.58]	0.06
True/false questions about risk factors of cognitive impairment							
a) Diseases such as hypertension and diabetes increase the risk of dementia	33	0.69	[0.56-0.82]	17	0.57	[0.39-0.75]	0.28
b) Smoking and drinking alcohol are not related to memory loss	30	0.63	[0.5-0.76]	18	0.6	[0.42-0.78]	0.79
c) Having relatives with dementia increases the risk of dementia	32	0.67	[0.54-0.80]	24	0.8	[0.66-0.94]	0.21
d) Frequent physical activity is not important	42	0.88	[0.73-1.03]	28	0.93	[0.84-1.02]	0.48
e) Medicines are available to prevent and cure Alzheimer's disease	32	0.67	[0.54-0.8]	20	0.67	[0.50-0.84]	1

n_1_:N_1_: number of participants in group 1; n_2_:N_2_: number of participants in group 2; proportion: proportion in each group; *significance. Z-test for proportions; **significant results.

**Figure 1.**
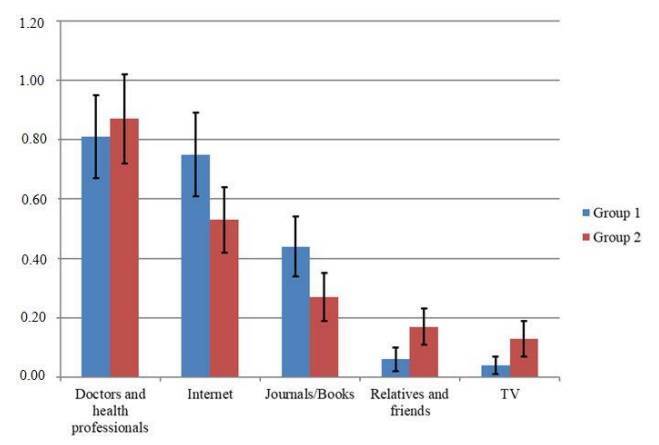
Sources sought for information about memory, dementia, and Alzheimer’s disease: proportion of answers. Question: “What is most important to get information about memory, dementia and Alzheimer’s disease?”- Answers: 1 - Doctors and health professionals (n_1_:N_1_=39; proportion=0.81; 95%CI 0.67-0.95 vs. n_2_:N_2_=26; proportion =0.87; 95%CI 0.72-1.02; p=0.49). 2 - Internet (n_1_:N_1_=36; proportion=0.75; 95%CI 0.61-0.89 vs. n_2_:N_2_=16; proportion=0.53; 95%CI 0.42-0.64; p=0.04). 3 - Journal/books (n_1_:N_1_=21; proportion=0.44; 95%CI 0.34-0.54 vs. n_2_:N_2_=8; proportion=0.27; 95%CI 0.19-0.35; p=0.13). 4 - Relatives and friends (n_1_:N_1_=3; proportion=0.06; 95%CI 0.02-0.1 vs. n_2_:N_2_=5; proportion=0.17; 95%CI 0.11-0.23; p=0.19). 5 - TV (n_1_:N_1_=2; proportion=0.04; 95%CI 0.01-0.07 vs. n_2_:N_2_=4; proportion=0.13; 95%CI 0.07-0.19; p=0.14). Z-test for proportions was used to obtain p values.

When the participants were asked about sources of information, the three most common answers were: *Doctors and health professionals* (n_1_:N_1_=39; proportion=0.81 *vs.* n_2_:N_2_=26; proportion=0.87); *Internet* (n_1_:N_1_=36; proportion=0.75 *vs.* n_2_:N_2_=16; proportion=0.53), and *Journal/books* for both groups (n_1_:N_1=_21; proportion=0.44 *vs.* n_2_:N_2_=8; proportion=0.27). Significant differences were found for *Internet* (p=0.04), as the participants in group 1 would use *the Internet* as first source of information, instead of asking *doctors or health professionals* (Figure 1).

### Cognitive impairment causes and risks factors

When asked *Which was the most common cause of memory loss in people over 65 years old?*, Alzheimer’s disease was identified correctly as the main cause by participants in both groups. The confidence intervals for proportions did not show significant differences between groups (Table 3).

Next, five true/false questions about causes and risk factors of cognitive impairment were presented (Table 3). As expected, the proportions of correct answers were higher in relatives of patients with cognitive impairment (group 1), but the results did not show statistical significance (Table 3).

The analysis of these results indicates a question that could be posed: if the relatives of patients with cognitive impairment have more self-initiative to be informed about dementia, *why don’t they have more knowledge about cognitive impairment?*. This might reflect deficiencies in the quality of the sources of information available.

## DISCUSSION

Despite the high prevalence of cognitive impairment and dementia, information about this syndrome is poor, especially among the general population.^5^ ^,^ ^12^ ^,^ ^16^ Many people do not know the characteristics of cognitive functions in older adults and assume that memory loss is related to aging, thus delaying the diagnosis of neurodegenerative diseases.^3^ ^,^ ^17^ Furthermore, there seems to be a lot of wrong perceptions and misinformation, which negatively impact not just the people with dementia and their caregivers, but also society, since it means that we are not ready to manage dementia and its consequences.^2^ ^,^ ^6^ ^,^ ^11^

A very common situation is the individual who receives a medical diagnosis and looks for information to understand his/her condition. Nowadays, the Internet has become an important source of health information for all people, especially patients and their relatives.^18^ In a national survey commented by McCarthy et al.,^18^ it was reported that, in 2012, around 72% of adult users sought health information on the Internet, with these searches being mainly about general health, but also related to an already diagnosed condition or treatment. In this survey, 35% of American adult users accessed online sources to determine what kind of clinical condition they possibly had, and 46% of these people felt they should seek medical attention on the basis of what they found online.^18^

In our study, as expected, the relatives of patients with cognitive impairment (group 1) had looked for information more frequently than the relatives of older adults without cognitive impairment (group 2) (n_1_:N_1_=31; proportion=0.65 [0.52‒0.78] *vs.* n_2_:N_2_=10; proportion=0.33 [0.24‒0.42] p≤0.01). It might be explained by the fact that almost half of the participants in group 1 were family caregivers, but also because they were close relatives of someone with cognitive impairment. However, in this study, being a caregiver did not seem to influence the self-initiative to be informed, though these results might be limited by the small size of the sample.

In group 2, it was seen that more than half of the participants knew someone with dementia, but they did not share the same interest. This might mean that being in contact with this syndrome leads the close relatives (especially those who assume the caregiver role) to take the initiative to be informed.

Dementia is one of the ten most common causes of disability in older adults,^2^ ^,^ ^3^ in our sample, 23 participants (proportion=0.48) were family caregivers, mainly females and first-degree relatives, which is consistent with reported findings about informal caregiving in dementia.^3^ ^,^ ^7^ ^,^ ^8^ On the other hand, among older patients without cognitive impairment, just 2 (proportion=0.06) needed a private caregiver, a non-relative person hired to help them with some activities at home.

When asked about the most important sources of information, the three most common answers in both groups were: *1- Doctors and health professionals*, *2- Internet*, and *3- Journal/books*. Curiously the most common answer for group 1 was *Internet* (p=0.04) instead of *doctor and health professionals*. There might be some variables to explain this behavior, such as urgency in being informed, as they have someone close who is suffering, as well as facilities and difficulties in accessing the health care system, difficulties in understanding the medical guidance, or even lack of medical counseling during the consultations. These findings highlight the need of assessing the quality of information about dementia available on the Internet and other sources, but also the necessity of ensuring that health professionals, especially those in primary care, be prepared to provide good medical advice about cognitive impairment and dementia.^3^

Despite the fact that the relatives of patients with cognitive impairment have more self-initiative to look for information about dementia, this does not seem to improve dementia knowledge.^5^ ^,^ ^8^ ^,^ ^12^ ^,^ ^13^ ^,^ ^16^ ^,^ ^17^ Low and Anstey^19^ studied dementia literacy, assessing the beliefs about causes, risk reduction, and prognosis among 2,000 randomly selected community-dwelling adults who answered a telephone survey. A total of 82% of the interviewees correctly identified “dementia” or “Alzheimer’s disease” from a vignette, but there were no differences in terms of recognition rates between the descriptions of mild or moderate dementia symptoms; the participants did not know the association between dementia and cardiovascular factors, and almost half of them thought that people with dementia could partially recover with appropriate treatment.

In our results, among the relatives of patients with cognitive impairment, there were 12 participants (proportion=0.25; n_1_:N_1_=12) who believed that memory loss is caused by normal aging; this proportion was higher among the relatives of older adults without cognitive impairment (proportion=0.4; n_2_:N_2_=12). This might mean that taking care of someone with dementia changes the concepts about memory and aging, but only in a small proportion.^5^ ^,^ ^9^ ^,^ ^13^ ^,^ ^17^ This also could indicate that deeply rooted beliefs may be hard to change, even when facing reality.^7^ ^,^ ^12^ When identifying risk factors, such as hypertension, diabetes, smoking, and family history, slightly more than half of the participants in both groups answered correctly. Moreover, almost a third of the participants believed that there are drugs to prevent and cure Alzheimer’s disease. These results indicate an illusory optimism about dementia and highlight the importance of promoting public awareness campaigns to improve dementia literacy.^3^ ^,^ ^6^ ^,^ ^7^ ^,^ ^8^ ^,^ ^19^

People’s beliefs and perceptions affect the way dementia is portrayed in society, which affects how society manages this problem and how each one acts towards people with dementia.^10^ These beliefs may be formed on the basis of education and information, but they are also influenced by moral and cultural aspects.^10^ ^,^ ^11^ ^,^ ^13^ ^,^ ^17^ In some rural communities of South Africa, for example, dementia is often believed as something connected to witchcraft, rather than to disease,^11^ and what is more unbelievable is that nurses also share this belief. In these communities, people with dementia are deemed as witches, and the inhabitants have negative reactions towards them; these patients are bullied, banished, burned, stoned, and even killed. This “witch belief” also prevents the patients and their relatives from seeking professional medical health.^11^

Planning informative interventions for educating people about cognitive impairment in aging and dementia is a good initiative to start changing the wrong perceptions and misinformation about this syndrome.^12^ ^,^ ^15^ Forbes et al.^9^ showed the effectiveness of educative interventions for supporting people with dementia, their caregivers, and also the health practitioners. This study identified six stages of the dementia care journey and allowed the caregivers and health care providers to identify these stages, as well as their needs in each of them. Interventions with this kind of support are very important to the caregivers, who often do not have the time, skills, energy or knowledge to seek information on their own.^6^ ^,^ ^7^ ^,^ ^9^ As people with dementia do not identify this need, caregivers are potentially alone in the dementia care journey.^8^ ^,^ ^9^

Some studies^8^ ^,^ ^13^ ^,^ ^15^ suggest the existence of high- and moderate-quality evidence reporting that psychosocial interventions are acceptable among the caregivers, and that these interventions are more likely to be accepted when caregivers recognize their needs and when intervention is offered by professionals with knowledge and experience on dementia care.^9^ ^,^ ^12^ As we have seen in our study, many relatives and caregivers, in both groups, were interested in participating in an educational program about cognitive impairment and dementia (n_1_:N_1_=43; proportion=0.89; *vs.* n_2_:N_2_=26; proportion=0.87).

Evidence suggests that the ability to deal with the needs of patients with dementia improves when the caregivers receive professional support during the illness process.^7^ ^,^ ^8^ ^,^ ^9^ ^,^ ^11^ ^,^ ^12^ ^,^ ^15^ ^,^ ^16^ Organizing and planning interventions for dementia literacy and support, especially among the caregivers, offered by trained professionals, should be a priority in public health systems.

This study had some limitations. The follow-up time of the patients was very variable, as well as the years of schooling of the participants. Only a few participants had a profession/occupation related to health care. It additional studies comparing the beliefs and knowledge of this specific group (health professional - relative/caregiver), it would be interesting to identify more variables that could influence dementia literacy. Studies with larger samples are needed to get more accurate values. We did not make distinctions between relatives and caregivers, because our objective was just to identify beliefs about dementia and aging among people in contact with older people with and without cognitive impairment, assuming that being a close relative of a dementia patient had an important influence on these beliefs.

Nevertheless, this study identified the need of improving dementia knowledge among the general population. Coping with ignorance and misinformation may help to develop effective strategies to prevent dementia and mitigate its impacts.^3^ ^,^ ^6^ ^,^ ^12^ The combination of some specific non-pharmacological interventions such as family counseling, informal caregiver’s education, music therapy, and other psychosocial interventions together with specific medications, could improve life quality for patients, caregivers, and patients’ relatives.^15^ Further research in this area is required to verify the quality of the evidence supporting these strategies.

Promoting education about dementia and risk/protective factors is needed, especially in the public health system, as educational interventions can motivate healthy changes in lifestyle to control risk factors and reduce the prevalence of dementia.^2^ ^,^ ^3^ ^,^ ^15^
